# Prognostic value of PANOMEN-3 classification for hypopituitarism in surgically and nonoperatively managed pituitary adenomas

**DOI:** 10.1530/EC-26-0123

**Published:** 2026-03-26

**Authors:** Afif Nakhleh, Gida Ayada, Mai Assalia Naffa, Gill Sviri, Sagit Zolotov

**Affiliations:** ^1^ Institute of Endocrinology, Diabetes and Metabolism, Rambam Health Care Campus, Haifa, Israel; ^2^ The Azrieli Faculty of Medicine, Bar-Ilan University, Safed, Israel; ^3^ Department of Neurosurgery, Rambam Health Care Campus, Haifa, Israel; ^4^ Ruth & Bruce Rappaport Faculty of Medicine, Technion, Israel Institute of Technology, Haifa, Israel

**Keywords:** hypopituitarism, PANOMEN-3, nonoperative management, pituitary adenoma, secondary adrenal insufficiency, secondary hypothyroidism, transsphenoidal surgery

## Abstract

**Background:**

The prognostic value of Pituitary Adenoma Nomenclature 3 (PANOMEN-3) for incident or persistent hypopituitarism remains uncertain. This study aimed to assess whether baseline PANOMEN-3 grade and its components predict secondary adrenal insufficiency or secondary hypothyroidism in surgically and nonoperatively managed patients.

**Methods:**

We conducted a retrospective cohort study of adults with newly diagnosed pituitary adenoma at Rambam Health Care Campus, a tertiary referral center in Haifa, Israel, between 2010 and 2023. Follow-up was censored on August 31, 2025. Patients were stratified by management (surgical or nonoperative). PANOMEN-3 grades were assigned at the index visit, defined as the initial endocrinology consultation. The primary outcome was the incidence of the composite of secondary adrenal insufficiency or secondary hypothyroidism at follow-up.

**Results:**

The study included 208 adults with pituitary adenomas: 118 underwent transsphenoidal surgery and 90 were managed nonoperatively. In the surgical group, the incidence of secondary adrenal insufficiency or secondary hypothyroidism at a median follow-up of 17.4 months (IQR: 5.0–47.7) was 32.2% and increased with higher baseline PANOMEN-3 grade (0% in grade 0 to 61.1% in grade 3; *P* = 0.01). In the nonoperative group, the incidence was 11.1% at a median follow-up of 15.5 months (IQR: 5.7–35.8) and was likewise associated with higher baseline grade (0% in grade 0 to 20.0% in grade 3; *P* = 0.02). Across both groups, baseline hypopituitarism and larger adenoma size were associated with secondary adrenal insufficiency or secondary hypothyroidism (surgical: *P* < 0.001 and *P* = 0.04; nonoperative: *P* < 0.001 and *P* = 0.03).

**Conclusion:**

PANOMEN-3 grading effectively stratifies the risk of secondary adrenal and thyroid dysfunction in both surgical and nonoperative settings.

## Introduction

Pituitary adenomas are the most common sellar masses in adults, accounting for approximately 15% of all intracranial neoplasms (1). Morbidity primarily results from hormone hypersecretion and the local mass effect within the sella turcica (1, 2). Although traditionally considered benign, rare tumors may exhibit aggressive behavior or malignant transformation (3).

Historically, pituitary adenomas were classified by size, hormone secretion, radiologic invasiveness, and histopathology. In 2013, a five-tiered clinical–pathological classification was proposed, based on invasiveness and proliferation (4). This approach presents challenges, given that over half of all adenomas do not necessitate surgical resection. Consequently, the majority of pituitary neoplasms cannot be effectively classified solely based on histopathological analysis (5). In 2022, the World Health Organization (WHO) redefined them as ‘pituitary neuroendocrine tumors’ (PitNETs) to reflect an updated understanding of their biology (6). Yet, the reliance on histopathology remains a barrier in routine practice for adenomas managed without surgery.

The Pituitary Adenoma Nomenclature 3 (PANOMEN-3) fills this gap by providing a purely clinical–radiological tool applicable to both operated and nonoperated patients. Introduced in 2024, this system integrates nine key parameters: hormonal phenotype, secretory status, hypopituitarism, adenoma size, mass effect, invasion, residual tumor, histopathology, and genetic syndromes (7). PANOMEN-3 generates a corrected composite score that stratifies tumors into four prognostic grades (grade 0 through grade 3). Radiological grading systems, such as the Knosp scale, further enhance prognostic accuracy (7).

The PANOMEN-3 clinicopathological classification appears to be a robust prognostic tool for pituitary adenomas across all treatment settings, including nonsurgical management. Recent observational studies suggest that the score stratifies prognosis effectively, as demonstrated by a large Spanish multicenter cohort (*n* = 1,143; 814 operated and 329 conservatively managed). In this cohort, PANOMEN-3 showed an accuracy of approximately 76% for predicting recurrence or progression over a median of 8.8 years (8). The score reflects stepwise risk increases from grade 0 to 3, with the most significant risk factors being residual tumor, hereditary syndromes, and active secretory status. This evidence strongly supports using the classification for both operated and conservatively managed patients.

Another single-center retrospective cohort study in the postoperative setting shows that higher PANOMEN-3 grades are associated with a shorter disease-free survival, a greater likelihood of residual mass or recurrence, and an earlier need for additional therapy (9). However, its performance may be weaker than the five-tier clinicopathological system in a direct comparison (4, 9).

The predictive value of the PANOMEN-3 score for incident or persistent hypopituitarism during follow-up is currently unclear. This study, therefore, aimed to assess whether the baseline PANOMEN-3 grade and its individual component risk factors are associated with secondary adrenal insufficiency or secondary hypothyroidism during follow-up in both surgically and nonoperatively managed patients.

## Materials and methods

### Study design and subjects

This retrospective cohort study was conducted at Rambam Health Care Campus (RHCC), a tertiary referral center in Haifa, Israel. We reviewed the electronic medical records of adults (≥18 years) who first presented with a pituitary adenoma, confirmed by magnetic resonance imaging (MRI), to the RHCC Endocrinology Clinic in our center between January 1, 2010, and December 31, 2023. The index date was the first documented endocrinology clinic visit during the study period for nonoperatively managed patients and the first endocrinology clinic visit before surgery for surgically treated patients. Follow-up extended from the index date to the last endocrinology clinic visit before data extraction (August 31, 2025).

Patients were included if they had at least one clinic visit occurring ≥3 months after the index visit. Patients were excluded if, at the index date, they had received prior medical or surgical treatment for their pituitary adenoma; presented acutely with pituitary apoplexy; had primary hypothyroidism, primary adrenal insufficiency, or congenital hypopituitarism; or had active malignancy.

### Study objectives

The primary objective was to compare the incidence of the composite endpoint of secondary adrenal insufficiency or secondary hypothyroidism during follow-up across baseline PANOMEN-3 grades in surgical and nonoperative groups. Secondary objectives were i) to compare the incidence of individual outcomes during follow-up across PANOMEN-3 grades, including secondary adrenal insufficiency, secondary hypothyroidism, arginine vasopressin (AVP) deficiency, secondary hypogonadism (in men or premenopausal women), visual field defects, cranial nerve palsies, repeat pituitary surgery, medical treatment use, pituitary radiosurgery, and mortality and ii) to assess associations between individual baseline PANOMEN-3 components and the composite endpoint at follow-up within each treatment group.

### Variables, definitions, and collection methods

We queried the RHCC electronic medical record to identify patients with pituitary adenoma. A free-text search using the terms ‘pituitary’ and ‘adenoma’ in both Hebrew and English was performed. We retrospectively abstracted data on clinical presentation, management, and outcomes for the identified patients. Baseline clinical and biochemical values were defined as the most recent results recorded within 365 days or 30 days after the index date. The baseline biochemical values for patients who underwent pituitary surgery were captured preoperatively.

Records from the initial (index) visit and the most recent clinic visit were reviewed to extract demographics, clinical history, presenting signs and symptoms, biochemical evaluations, neuro-ophthalmologic assessments, imaging findings, management approach, pathological findings (for surgically managed patients), and details of medical therapy. The PANOMEN-3 grade was assigned at the index visit, in accordance with the previously published classification system (7). For both surgically and nonoperatively treated groups, PANOMEN-3 raw scores were mapped to grades as follows: grade 0 (raw = 0; corrected score = 0), grade 1 (raw = 1–2; 0 < corrected score < 0.30), grade 2 (raw = 3–4; 0.30 ≤ corrected score ≤ 0.60), and grade 3 (raw ≥ 5; corrected score > 0.60). The denominator for the corrected score calculation was 7 (hormonal phenotype, secretory status, hypopituitarism, tumor size, mass effect, invasion, and genetic syndromes) (7). For surgically treated individuals, the PANOMEN-3 score was calculated preoperatively.

We extracted age, sex, weight, body mass index (BMI), and blood pressure. Clinical histories were reviewed for comorbidities, including hypertension, hyperlipidemia, diabetes mellitus, cardiovascular disease, and chronic kidney disease.

Biochemical evaluation of pituitary function included serum cortisol, adrenocorticotropic hormone (ACTH), thyroid-stimulating hormone (TSH), free thyroxin (FT4), prolactin, luteinizing hormone (LH), follicle-stimulating hormone (FSH), growth hormone (GH), age-adjusted insulin-like growth factor 1 (IGF-1), and total testosterone (in men). Sex-steroid assays also included estradiol in women. Additional laboratory tests abstracted from the medical record included sodium, plasma osmolarity, and urine osmolality, as available. Results of the Synacthen stimulation test were recorded where performed.

Secondary adrenal insufficiency was defined by random serum cortisol <80 nmol/L or 60-min cortisol <500 nmol/L after 250-mcg Synacthen, together with low or inappropriately normal ACTH. Secondary hypothyroidism was defined as FT4 below the normal range, together with low or inappropriately normal TSH. Gonadotropic deficiency in men was defined as low testosterone levels with low or inappropriately normal gonadotrophins. Gonadotropic deficiency in postmenopausal women was defined as inappropriately low gonadotropins for menopausal age. In premenopausal women, gonadal function was assessed clinically and biochemically, specifically requiring low serum estradiol with evidence of oligomenorrhea or amenorrhea.

At baseline, we defined hypercortisolism using 24-h urinary free cortisol (UFC) and 8:00 AM serum cortisol after a 1-mg dexamethasone suppression test. During follow-up, hypercortisolism was defined by 24-h UFC.

Pituitary adenoma classification was based on functional status. Non-functioning adenomas were defined by i) a pituitary mass on MRI and ii) the absence of hormonal overproduction, specifically no elevated serum prolactin (>2,000 mU/L), IGF-1, or FT4 and no clinical suspicion of Cushing’s disease in the medical records. Functioning adenomas were identified via ICD-9 diagnosis codes and validated by a manual review of biochemical data to ensure that they met diagnostic thresholds. A prolactinoma diagnosis required marked hyperprolactinemia (>2,000 mU/L) at the initial visit. The diagnosis of acromegaly was confirmed by an IGF-I level greater than 1.3 times the upper limit of normal for age in patients with typical clinical phenotypes. Cushing’s disease was confirmed by elevated 24-h UFC and/or failure to suppress serum cortisol to <50 nmol/L after a 1-mg overnight dexamethasone suppression test, accompanied by typical clinical symptoms and signs as confirmed by a review of medical charts. TSH-secreting adenomas were defined by elevated FT4 with non-suppressed TSH and the presence of consistent clinical features documented in the medical record.

Pituitary dysfunction at the last follow-up visit was identified using ICD-9 codes and confirmed by the requirement for hormone replacement (a prescription filled within 90 days of the last visit).

Ophthalmologic complications, including visual field defects and cranial nerve palsies, were recorded. MRI findings were reported by certified neuroradiologists. These findings were specifically reviewed for adenoma size, mass effect, and evidence of local invasion.

Management data included the overall treatment approach and, for surgically managed patients, pathological findings. Surgical management consisted of the endoscopic endonasal approach, which is the standard of care at our institution. Surgical records documented transsphenoidal procedures, reoperations, and radiosurgery. Cerebrospinal fluid leak was recorded when present. Histopathology was retrieved when available. Medication use was documented, including hormone replacement therapies (corticosteroids, levothyroxine, sex steroids, and desmopressin), dopamine agonists and somatostatin analogs, and GH receptor antagonists.

The study received ethical approval from our institutional review board (September 23, 2024). Informed consent was waived for this study because of its observational design.

### Statistical analysis

Categorical variables were reported as counts and percentages, and continuous variables were reported as means with standard deviations or medians with interquartile ranges. Comparisons across PANOMEN-3 grades utilized Fisher’s exact test or the chi-square test for categorical variables and the Mann–Whitney *U* test or the Kruskal–Wallis test for continuous variables. Two-sided *P* values < 0.05 were considered statistically significant. All analyses were conducted in IBM SPSS Statistics, version 25 (IBM Corp., USA).

## Results

**Figure 1:**
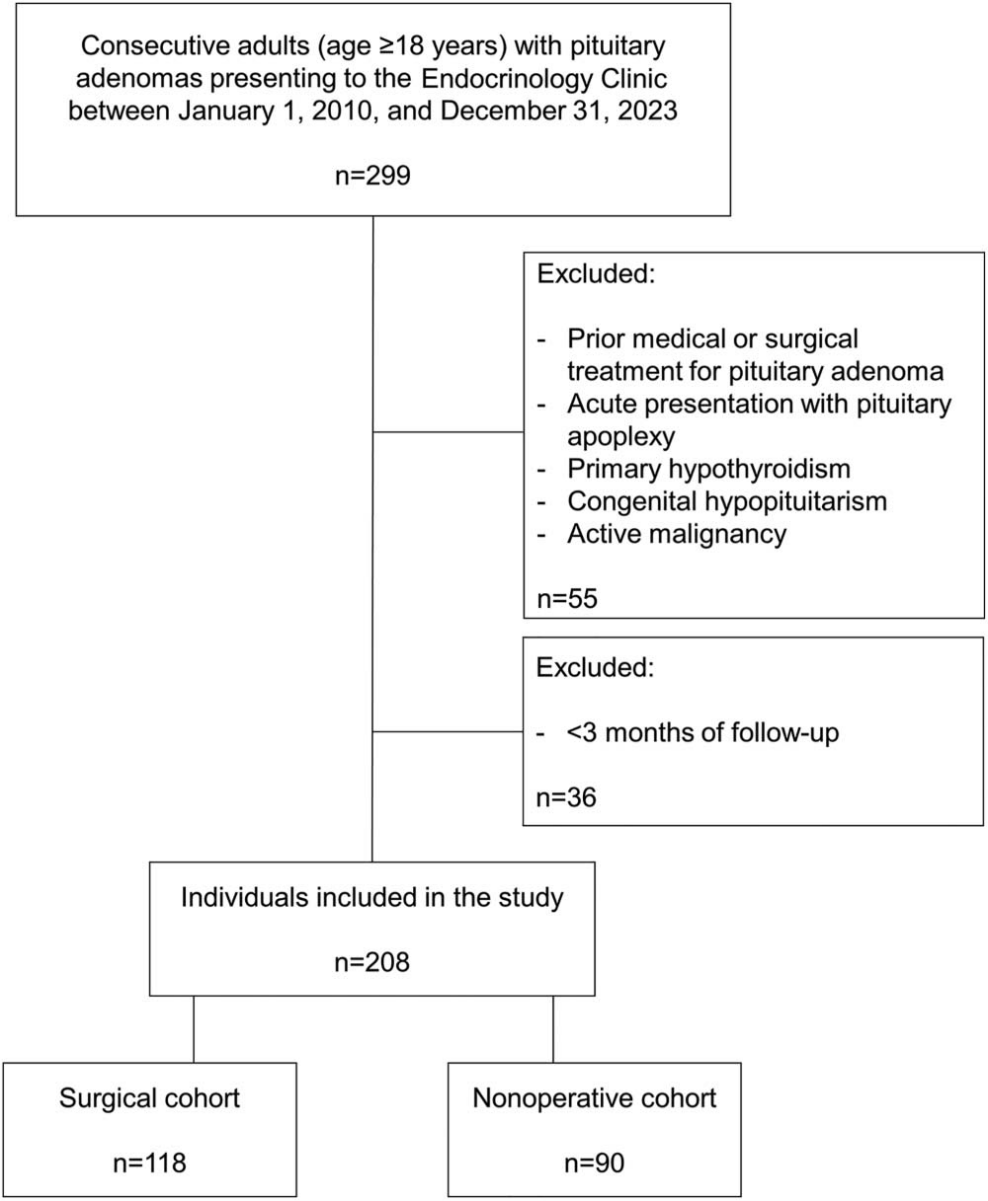
Flow diagram of individuals included in the study.

The study included 208 individuals with pituitary adenomas: 118 underwent transsphenoidal surgery, and 90 were managed nonoperatively (Fig. 1).

In the surgical group, the median age was 52.3 years (IQR: 40.3–64.0), and 54.2% were male (Table 1). PANOMEN-3 grades were significantly associated with a greater proportion of functioning adenomas (0% in grade 0 vs 55.6% in grade 3; *P* < 0.001), larger tumors (all grade 0 patients had microadenomas, whereas 66.7% of grade 3 patients had giant adenomas; *P* < 0.001), more frequent Knosp 3–4 invasion (77.8% in grade 3; *P* < 0.001), and more common preoperative secondary adrenal insufficiency (38.9% in grade 3; *P* = 0.002). Optic chiasm abutment showed a trend toward higher frequency in higher grades (*P* = 0.06); however, preoperative visual field defects did not differ across grades. Age, sex, and BMI were comparable across grades. In addition, there were no significant differences regarding preoperative secondary hypothyroidism, AVP deficiency, or secondary hypogonadism.

**Table 1:** Baseline characteristics of individuals with pituitary adenomas treated surgically. Data are presented as median (IQR) or as *n* (%).

	Total *n* = 118	PANOMEN-3 grade 0: *n* = 2 (1.7%)	PANOMEN-3 grade 1: *n* = 58 (49.2%)	PANOMEN-3 grade 2: *n* = 40 (33.9%)	PANOMEN-3 grade 3: *n* = 18 (15.3%)	*P* value
Age, years	52.3 (40.3–64.0)	51.7 (43.9–59.6)	54.4 (41.6–67.1)	49.0 (32.2–58.0)	48.7 (43.53–63.7)	0.33
Male sex	64 (54.2)	1 (50.0)	31 (53.5)	21 (52.5)	11 (61.1)	0.91
BMI (kg/m^2^)	27.8 (24.7–31.4)	29.3 (28.4–30.1)	26.2 (24.6–31.9)	27.9 (23.9–29.7)	27.8 (25.2–32.6)	0.84
Diabetes mellitus	31 (26.7)	1 (50.0)	14 (24.1)	13 (32.5)	3 (16.7)	0.41
Hypertension	41 (34.8)	0 (0.0)	21 (36.2)	13 (32.5)	7 (38.9)	0.87
Hyperlipidemia	39 (33.1)	1 (50.0)	17 (29.3)	13 (32.5)	8 (44.4)	0.54
Cardiovascular disease	12 (10.2)	0 (0)	5 (8.6)	4 (10.0)	3 (16.7)	0.71
Chronic renal failure	2 (1.72)	0 (0)	2 (3.5)	0 (0)	0 (0)	0.66
Functioning adenoma^‡^	39 (33.1)	0 (0)	10 (17.2)	19 (47.5)	10 (55.6)	<0.001
Prolactinoma^$^	15 (38.5)	0 (0)	4 (40.0)	6 (31.6)	5 (50.0)	0.40
Acromegaly^$^	19 (48.7)	0 (0)	4 (40.0)	12 (63.2)	3 (30.0)	
Cushing’s disease^$^	4 (10.3)	0 (0)	1 (10.0)	1 (5.3)	2 (20.0)	
Thyrotropin-secreting adenoma^$^	1 (2.6)	0 (0)	1 (10.0)	0 (0)	0 (0)	
Adenoma size*						
Microadenoma	22 (18.6)	2 (100.0)	15 (25.9)	5 (12.5)	0 (0)	<0.001
Macroadenoma	70 (59.3)	0 (0)	36 (62.1)	28 (70.0)	6 (33.3)	
Giant adenoma	26 (22.1)	0 (0)	7 (12.0)	7 (17.5)	12 (66.7)	
Cavernous sinus invasion (Knosp 3–4)	44 (37.3)	0 (0)	10 (17.2)	20 (50.0)	14 (77.8)	<0.001
Optic chiasm abutment	57 (48.3)	0 (0)	28 (48.3)	16 (40.0)	13 (72.2)	0.06
Preoperative visual field defect	38 (34.6)	0 (0)	17 (31.5)	11 (30.6)	10 (55.6)	0.20
Preoperative cranial nerve palsy	2 (1.7)	0 (0)	1 (1.7)	0 (0)	1 (5.6)	0.42
Preoperative CSF leak	1 (0.9)	0 (0)	1 (1.7)	0 (0)	0 (0)	1.0
Preoperative secondary AI	23 (19.5)	0 (0)	4 (6.9)	12 (30.0)	7 (38.9)	0.002
Preoperative secondary HT	26 (22.0)	0 (0)	9 (15.5)	11 (27.5)	6 (33.3)	0.26
Preoperative AVP deficiency	4 (3.4)	0 (0)	0 (0)	4 (10.0)	0 (0)	0.07
Preoperative secondary HG	12 (10.2)	0 (0)	3 (5.2)	6 (15.0)	3 (16.7)	0.24
Increased proliferation (Ki-67 > 10%)	2 (1.7)	0 (0)	0 (0)	0 (0)	2 (11.1)	0.06
High-risk subtype^†^	9 (7.6)	0 (0)	2 (3.4)	4 (10)	3 (16.7)	0.22

AVP, arginine vasopressin; BMI, body mass index; and CSF, cerebrospinal fluid; AI, adrenal insufficiency; HT, hypothyroidism; HG, hypogonadisim.

* Adenoma size criteria were defined as follows: microadenoma, diameter <1 cm; macroadenoma, diameter ≥ 1 cm and <4 cm; and giant adenoma, diameter ≥ 4 cm.

^†^ High-risk subtypes include null cell adenoma and sparsely granulated somatotroph adenoma.

^$^ *n* (%) of functioning.

^‡^ Biochemically active at baseline.

In the nonoperative group, the median age was 39.2 years (IQR: 24.9–55.8), and 46.7% were male (Table 2). Higher PANOMEN-3 grades were significantly associated with older age (*P* = 0.04), male sex (*P* = 0.02), and higher BMI (*P* = 0.003). The prevalence of functioning adenomas (97.2% of which were prolactinomas) and larger adenoma size also increased with higher grades (both *P* < 0.001). Consequently, optic chiasm abutment (*P* = 0.003) and visual field defects (*P* = 0.03) were more frequent in higher grades, as were baseline secondary adrenal insufficiency (*P* = 0.03) and secondary hypogonadism (*P* < 0.001). Baseline secondary hypothyroidism and AVP deficiency did not differ significantly across grades.

**Table 2:** Baseline characteristics of individuals with pituitary adenomas treated nonoperatively. Data are presented as median (IQR) or as* n *(%).

	Total *n* = 90	PANOMEN-3 grade 0: *n* = 29 (32.2%)	PANOMEN-3 grade 1: *n* = 31 (34.4%)	PANOMEN-3 grade 2: *n* = 25 (27.8%)	PANOMEN-3 grade 3: *n* = 5 (5.6%)	*P* value
Age, years	39.2 (24.9–55.8)	29.2 (19.3–50.3)	37.0 (24.9–62.6)	47.3 (35.5–56.7)	41.78 (36.3–55.8)	0.04
Male sex	42 (46.7)	9 (31.0)	14 (45.2)	14 (56.0)	5 (100.0)	0.02
BMI (kg/m^2^)	29.1 (23.8–32.4)	23.6 (21.5–29.0)	29.2 (25.2–32.7)	30.2 (29.4–33.3)	32.8 (28.0–36.0)	0.003
Diabetes mellitus	13 (14.4)	3 (10.3)	3 (9.7)	7 (28.0)	0 (0)	0.19
Hypertension	25 (27.8)	7 (24.1)	9 (29.0)	9 (36.0)	0 (0)	0.48
Hyperlipidemia	18 (20.0)	3 (10.3)	7 (22.6)	6 (24.0)	2 (40.0)	0.27
Cardiovascular disease	7 (7.8)	2 (6.9)	2 (6.5)	3 (12.0)	0 (0.0)	0.86
Chronic renal failure	2 (2.2)	0 (0)	0 (0)	2 (8.0)	0 (0)	0.18
Functioning adenoma^‡^	36 (40.0)	0 (0)	13 (41.9)	18 (72.0)	5 (100.0)	<0.001
Prolactinoma^†^	35 (97.2)	0 (0)	12 (92.3)	18 (100.0)	5 (100.0)	0.50
Acromegaly^†^	1 (2.8)	0 (0)	1 (7.7)	0 (0)	0 (0)	
Cushing’s disease^†^	0 (0)	0 (0)	0 (0)	0 (0)	0 (0)	
Thyrotropin-secreting adenoma^†^	0 (0)	0 (0)	0 (0)	0 (0)	0 (0)	
Adenoma size*						
Microadenoma	51 (56.7)	29 (100.0)	19 (61.3)	3 (12.0)	0 (0)	<0.001
Macroadenoma	32 (35.6)	0 (0)	12 (38.7)	20 (80.0)	0 (0)	
Giant adenoma	7 (7.8)	0 (0)	0 (0)	2 (8.0)	5 (100.0)	
Cavernous sinus invasion (Knosp 3–4)	1 (1.1)	0 (0)	0 (0)	1 (4.0)	0 (0)	0.33
Optic chiasm abutment	14 (15.6)	1 (3.5)	3 (9.7)	7 (28.0)	3 (60.0)	0.003
Visual field defect	10 (19.2)	0 (0)	2 (11.8)	5 (26.3)	3 (60.0)	0.03
Cranial nerve palsy	0 (0)	0 (0)	0 (0)	0 (0)	0 (0)	—
CSF leak	0 (0)	0 (0)	0 (0)	0 (0)	0 (0)	—
Secondary AI	6 (6.7)	0 (0)	1 (3.2)	4 (16.7)	1 (20.0)	0.03
Secondary HT	7 (7.9)	0 (0)	3 (9.7)	3 (12.5)	1 (20.0)	0.12
AVP deficiency	1 (1.1)	0 (0)	0 (0)	1 (4.2)	0 (0)	0.33
Secondary HG	16 (18.0)	0 (0)	4 (12.9)	10 (41.7)	2 (40.0)	<0.001

AVP, arginine vasopressin; BMI, body mass index; and CSF, cerebrospinal flui; AI, adrenal insufficiency; HT, hypothyroidism; HG, hypogonadism.

* Adenoma size criteria were defined as follows: microadenoma, diameter <1 cm; macroadenoma, diameter ≥1 cm and <4 cm; and giant adenoma, diameter ≥4 cm.

^†^ *n* (%) of functioning.

^‡^ Biochemically active at baseline.

In both groups, other comorbidities (diabetes, hypertension, hyperlipidemia, and cardiovascular disease) did not differ significantly by grade. No patients in either group had a documented genetic syndrome.

The median follow-up was 17.4 months (5.0–47.7) in the surgical group and 15.5 months (5.7–35.8) in the nonoperative group.

At the last follow-up in the surgical group, any new secondary adrenal insufficiency or hypothyroidism was present in 32.2% of patients and was associated with higher baseline PANOMEN-3 grade (0% in grade 0;22.4% in grade 1; 35.0% in grade 2; 61.1% in grade 3;*P* = 0.01) (Table 3). Secondary adrenal insufficiency was observed in 22.9% (38.9% in grade 3; *P* = 0.15), and secondary hypothyroidism was observed in 21.2% (44.4% in grade 3; *P* = 0.05, borderline significance). AVP deficiency was present in 6.8% overall, and secondary hypogonadism was present in 18.7% of males and premenopausal females; neither varied significantly across PANOMEN-3 grades.

**Table 3:** Clinical outcomes at follow-up of individuals treated surgically. Data are presented as median (IQR) or as *n* (%).

	Total *n* = 118	PANOMEN-3 grade 0: *n* = 2 (1.7%)	PANOMEN-3 grade 1: *n* = 58 (49.2%)	PANOMEN-3 grade 2: *n* = 40 (33.9%)	PANOMEN-3 grade 3: *n* = 18 (15.3%)	*P* value
Follow-up, months	17.4 (5.0–47.7)	3.6 (3.1–4.1)	14.2 (3.8–41.5)	24.3 (5.8–58.8)	33.4 (6.0–41.4)	0.18
Residual tumor	63 (53.4)	0 (0)	6 (10.3)	39 (97.5)	18 (100)	<0.001
Prolactin elevated*^,^ ^‡^	9 (60)	0 (0)	1 (25.0)	3 (50.0)	5 (100.0)	0.07
IGF-1 elevated*^,^^$^	13 (68.4)	0 (0)	2 (50.0)	9 (75.0)	2 (66.6)	0.78
24-h UFC elevated*^,^^£^	1 (25)	0 (0)	0 (0)	0 (0)	1 (50)	1.0
Dopamine agonist use	9 (7.6)	0 (0)	3 (5.2)	3 (7.5)	3 (16.7)	0.39
Somatostatin analog use	11 (9.3)	0 (0)	1 (1.7)	7 (17.5)	3 (16.7)	0.04
GH receptor antagonist use	3 (2.5)	0 (0)	0 (0)	2 (5)	1 (5.6)	0.36
Repeat pituitary surgery	6 (5.1)	0 (0)	0 (0)	2 (5)	4 (22.2)	0.004
Pituitary radiosurgery	7 (5.9)	0 (0)	1 (1.7)	4 (10.0)	2 (11.1)	0.25
Visual field defect	9 (7.6)	0 (0)	4 (6.9)	1 (2.5)	4 (22.2)	0.10
Cranial nerve palsy	2 (1.7)	0 (0)	0 (0)	2 (5.0)	0 (0)	0.27
Secondary AI or HT	38 (32.2)	0 (0)	13 (22.4)	14 (35.0)	11 (61.1)	0.01
Secondary AI,	27 (22.9)	0 (0)	9 (15.5)	11 (27.5)	7 (38.9)	0.15
Secondary HT	25 (21.2)	0 (0)	8 (13.8)	9 (22.5)	9 (44.4)	0.05
AVP deficiency	8 (6.8)	0 (0)	3 (5.2)	5 (12.5)	0 (0)	0.34
Secondary HG, *n*	17 (18.7), 91	0 (0), 1	6 (14.0), 43	5 (16.1), 31	6 (37.5), 16	0.22
Mortality	6 (5.1)	0 (0.0)	4 (6.9)	2 (5.0)	0 (0.0)	0.87

AVP, arginine vasopressin; AI, adrenal insufficiency; HT, hypothyroidism; HG, hypogonadism; GH, growth hormone; IGF-1, insulin-like growth factor-1; and UFC, urinary free cortisol.

* Assessed 3–6 months post-op; all other outcomes at the last follow-up.

^†^ Men or premenopausal women.

^‡^ *n* (%) of prolactinoma cases.

^$^ *n* (%) of acromegaly cases.

^£^ *n* (%) of Cushing’s disease cases.

Higher PANOMEN-3 grades were also associated with the presence of a residual tumor (*P* < 0.001), the need for somatostatin analog therapy (*P* = 0.04), and repeat pituitary surgery (*P* = 0.004) (Table 3). No difference in mortality was observed.

In the nonoperative group, the composite of secondary adrenal insufficiency or secondary hypothyroidism was observed in 11.1% of patients at the last follow-up. The incidence was significantly associated with higher baseline PANOMEN-3 grades (0% in grade 0; 9.7% in grade 1; 24.0% in grade 2; 20.0% in grade 3; *P* = 0.02) (Table 4). Secondary adrenal insufficiency was observed in 8.9%, and secondary hypothyroidism was observed in 6.7%; both increased significantly with PANOMEN-3 grade (*P* = 0.03 for each). AVP deficiency was present in 3.3% overall, and secondary hypogonadism was present in 9.1% of males and premenopausal females; neither varied significantly across PANOMEN-3 grades (*P* = 0.32 and *P* = 0.22, respectively). Higher PANOMEN-3 grades were also associated with a greater likelihood of visual field defects at the last follow-up (*P* = 0.02), increased dopamine agonist use (*P* < 0.001), and higher mortality (*P* = 0.01).

**Table 4:** Clinical outcomes at follow-up of individuals treated nonoperatively. Data are presented as median (IQR) or as *n* (%).

	Total *n* = 90	PANOMEN-3 grade 0: *n* = 29 (32.2%)	PANOMEN-3 grade 1: *n* = 31 (34.4%)	PANOMEN-3 grade 2: *n* = 25 (27.8%)	PANOMEN-3 grade 3: *n* = 5 (5.6%)	*P* value
Follow-up, months	15.5 (5.7–35.8)	11.9 (5.6–20.4)	15.4 (4.3–29.0)	24.1 (7.2–114.2)	89.6 (62.1–94.5)	0.005
Visible adenoma at the last follow-up	65 (72.2)	21 (72.4)	22 (71.0)	17 (68.0)	5 (100.0)	0.58
Prolactin elevated*^,^ ^‡^	3 (8.8)	0 (0)	2 (16.6)	1 (5.9)	0 (0)	0.73
IGF-1 elevated*^,^ ^$^	0 (0)	0 (0)	0 (0)	0 (0)	0 (0)	—
Dopamine agonist use	33 (36.7)	0 (0)	11 (35.5)	18 (72.0)	4 (89.0)	<0.001
Somatostatin analog use	1 (1.1)	0 (0)	1 (3.2)	0 (0)	0 (0)	1.0
Pituitary radiosurgery	0 (0)	0 (0)	0 (0)	0 (0)	0 (0)	—
Visual field defect	3 (3.4)	0 (0)	0 (0)	2 (8.0)	1 (20.0)	0.02
Cranial nerve palsy	1 (1.2)	0 (0)	0 (0)	1 (4.0)	0 (0)	0.35
Secondary AI or HT	10 (11.1)	0 (0)	3 (9.7)	6 (24.0)	1 (20.0)	0.02
Secondary AI	8 (8.9)	0 (0)	2 (6.5)	5 (20.0)	1 (20.0)	0.03
Secondary HT	6 (6.7)	0 (0)	1 (3.2)	5 (20.0)	0 (0)	0.03
AVP deficiency	3 (3.3)	1 (3.5)	0 (0.0)	2 (8.0)	0 (0.0)	0.32
Secondary HG^†^, *n*	7 (9.1), 77	0 (0), 23	1 (3.9), 26	5 (21.7), 23	1 (20.0), 5	0.22
Mortality	4 (4.4)	0 (0.0)	1 (3.2)	2 (8.0)	1 (20.0)	0.01

AVP, arginine vasopressin; GH, growth hormone; IGF-1, insulin-like growth factor-1; and UFC, urinary free cortisol; AI, adrenal insufficiency; HT, hypothyroidism; HG, hypogonadism.

* Assessed at the last follow-up.

^†^ Men or premenopausal women.

^‡^ *n* (%) of prlactinoma cases.

^$^ *n* (%) of acromegaly cases.

In the surgical group (Table 5), analysis of individual risk factors showed that preexisting hypopituitarism (*P* < 0.001) and larger baseline adenoma size (*P* = 0.04) were associated with secondary adrenal insufficiency or hypothyroidism at the last follow-up. Stratified by baseline pituitary status, event rates were 13/76 for no hypopituitarism, 9/17 for partial hypopituitarism without adrenal/AVP deficiency, and 16/25 for adrenal/AVP deficiency.

**Table 5:** PANOMEN-3 risk factors, stratified by the presence of secondary adrenal insufficiency (AI) or hypothyroidism (HT) at follow-up, among surgically treated individuals. Data are presented as *n* (%).

	Secondary AI or HT		*P* value
	No: *n* = 80	Yes: *n* = 38	
Phenotype			
Non-functioning pituitary adenoma or prolactinoma	63 (78.7)	31 (81.6)	0.58
Acromegaly or thyrotropin-secreting adenoma	15 (18.8)	5 (13.2)	
Cushing’s disease	2 (2.5)	2 (5.3)	
Secretory status at baseline			
Elevated	28 (35.0)	11 (29.0)	0.54
Hypopituitarism			
Absent	63 (78.8)	13 (34.2)	<0.001
Partial without adrenal insufficiency or AVP deficiency	8 (10.0)	9 (23.7)	
Adrenal insufficiency or AVP deficiency	9 (11.3)	16 (42.1)	
Size*			
Microadenoma	17 (21.3)	5 (13.2)	0.04
Macroadenoma	51 (63.8)	19 (50.0)	
Giant adenoma	12 (15.0)	14 (36.8)	
Mass effect (visual field defect, cranial nerve palsy, or CSF leak)	20 (25.0)	13 (34.2)	0.38
Cavernous sinus invasion (Knosp 3–4)	27 (33.8)	17 (44.7)	0.31
Residual tumor	42 (52.5)	21 (55.3)	0.85
Histopathology (Ki-67 > 10% or high-risk subtype^†^)	7 (8.7)	4 (10.5)	0.74

AVP, arginine vasopressin; CSF, cerebrospinal fluid.

* Adenoma size criteria were defined as follows: microadenoma, diameter <1 cm; macroadenoma, diameter ≥1 cm and <4 cm; and giant adenoma, diameter ≥4 cm.

^†^ High-risk subtypes include null cell adenoma and sparsely granulated somatotroph adenoma.

In the nonoperative group (Table 6), secondary adrenal insufficiency or hypothyroidism at the last follow-up was significantly associated with baseline hypopituitarism (*P* < 0.001), larger adenoma size (*P* = 0.03), and the presence of mass effect (visual field defect) (*P* = 0.01). Stratified by baseline pituitary status, event rates were 1/63 for no hypopituitarism, 3/21 for partial hypopituitarism without adrenal/AVP deficiency, and 6/6 for adrenal/AVP deficiency.

**Table 6:** PANOMEN-3 risk factors, stratified by the presence of secondary adrenal insufficiency (AI) or hypothyroidism (HT) at follow-up, among nonoperatively treated individuals. Data are presented as *n *(%).

	Secondary AI or HT		*P* value
	No: *n* = 80	Yes: *n* = 10	
Phenotype			
Non-functioning pituitary adenoma or prolactinoma	79 (98.8)	10 (100.0)	1.0
Acromegaly	1 (1.2)	0 (0)	
Secretory status at baseline			
Elevated	33 (41.3)	3 (30.0)	0.73
Hypopituitarism			
Absent	62 (77.5)	1 (10.0)	<0.001
Partial without adrenal insufficiency or AVP deficiency	18 (22.5)	3 (30.0)	
Adrenal insufficiency or AVP deficiency	0 (0)	6 (60.0)	
Size*			
Microadenoma	49 (61.3)	2 (20.0)	0.03
Macroadenoma	26 (32.5)	6 (60.0)	
Giant adenoma	5 (6.3)	2 (20.0)	
Mass effect (visual field defect)	6 (7.5)	4 (40.0)	0.01
Cavernous sinus invasion (Knosp 3–4)	0 (0.0)	1 (10.0)	0.11

AVP, arginine vasopressin; CSF, cerebrospinal fluid.

* Adenoma size criteria were defined as follows – microadenoma: diameter <1 cm; macroadenoma: diameter ≥1 cm and <4 cm; giant adenoma: diameter ≥4 cm.

## Discussion

In this single-center cohort of 208 adults with newly diagnosed pituitary adenomas, higher baseline PANOMEN-3 grades were associated with an increased incidence of secondary adrenal and thyroid dysfunction at follow-up in both surgical and nonoperative groups. In particular, the composite endpoint (secondary adrenal insufficiency or secondary hypothyroidism) increased substantially from 0% in grade 0 to 61.1% in grade 3 among surgically managed patients and from 0 to 20.0% in the nonoperative group. Across both cohorts, baseline hypopituitarism and adenoma size were the dominant drivers of risk. In addition, clinical mass effect (visual field defect) was identified as a risk factor specifically in nonoperatively managed patients. This work extends prior studies validating PANOMEN-3 primarily for adenoma behavior and prognosis by demonstrating its prognostic value for endocrine outcomes (8, 9, 10).

Our findings align with and help contextualize several strands of prior evidence. In particular, MRI-based and size-based predictors of central adrenal or thyroid failure in non-functioning pituitary adenomas have been described. Vertical height, largest diameter, volume, and chiasmal compression were all associated with secondary adrenal insufficiency and hypothyroidism (11). Second, natural-history data show that new-onset hypopituitarism is uncommon during surveillance of non-functioning pituitary microadenomas (∼0.6%), and growth is infrequent and slow. This supports de-escalating imaging and hormonal retesting in the absence of growth or symptoms (12), which is broadly consistent with an earlier meta-analysis that found low overall event rates but higher risk in macroadenomas and solid lesions (13). Third, surgical series consistently report that axis recovery is at least as common as new deficits; however, the cortisol axis is the least likely to recover, with any new deficits occurring in ∼10–14% overall (14, 15). Finally, a recent risk prediction study across modalities found that treatment type, baseline hypopituitarism, macroadenoma, age > 50, and cavernous sinus invasion independently predict new hypopituitarism with good discrimination (16).

Importantly, our analysis supports the application of PANOMEN-3 as a ‘pan-tumor’ prognostic tool. Stratification by phenotype revealed no significant difference in the incidence of secondary adrenal or thyroid dysfunction in either the surgical (*P* = 0.58) or nonoperative (*P* = 1.0) group (Tables 5 and 6). This suggests that for these axes, the prognostic risk is not primarily driven by the specific hormonal subtype. A key strength of this study is the parallel analysis of surgical and nonoperative pathways, which reflects real-world management strategies. Furthermore, we utilized the standardized PANOMEN-3 score; this is readily assignable at index presentation and provides a comprehensive, objective framework for risk assessment. A higher baseline PANOMEN-3 grade effectively stratifies the risk of secondary adrenal or thyroid insufficiency in both surgical and conservative cohorts. This risk is largely driven by baseline hypopituitarism and adenoma size, mirroring established MRI and clinical thresholds (11, 12, 13, 14). While the individual components of the score (e.g. size and invasion) are established predictors of outcome, the utility of PANOMEN-3 lies in its ability to integrate these factors into a single stratifiable grade. This provides a practical framework to guide the intensity of hormonal surveillance and preoperative counseling, independent of the surgical approach selected. In nonoperative patients, the association with visual field defects aligns with chiasmal compression as a practical marker of endocrine vulnerability (11, 12).

In the surgical group, the incidence of the composite endpoint among patients with intact pituitary function at baseline was 17.1% (13/76) (Table 5), which is slightly higher than the 10–14% range generally reported in surgical series (14, 15). Conversely, patients presenting with baseline pituitary compromise demonstrated substantially higher rates of the composite outcome at follow-up (52.9% for partial deficits and 64.0% for severe deficits), consistent with predominant disease persistence or progression. A similar trend was observed in the nonoperative group (Table 6), where the outcome was rare in patients with intact baseline function (1.6%) but frequent in those with baseline deficits. Notably, only 5.9% of this surgical cohort received radiotherapy, a modality known to confer the greatest risk of long-term hypopituitarism (16).

Conversely, the incidence of AVP deficiency and secondary hypogonadism did not vary by PANOMEN-3 grade. This aligns with the existing literature, which suggests that post-transsphenoidal AVP deficiency is driven primarily by intraoperative factors, such as pituitary stalk manipulation, rather than preoperative tumor characteristics. Reported rates are consequently variable, though generally low (∼2%) (17, 18). Similarly, hypogonadism showed no clear association with grade, likely reflecting the competing effects of surgery and the multifactorial nature of gonadal dysfunction. Prior series suggest that hypogonadism has a complex, non-linear relationship with tumor size and sex and that postoperative recovery and deficit patterns are heterogeneous (19, 20).

Limitations include the retrospective and single-center design. We utilized a composite primary endpoint of secondary adrenal insufficiency or secondary hypothyroidism. We did not utilize ‘overall hypopituitarism’ as a primary endpoint because the assessment of secondary hypogonadism during follow-up was restricted to men and premenopausal women; including it would have necessitated the exclusion of postmenopausal women, who comprised a significant portion of the cohort. Results are also subject to potential misclassification due to variability in clinical biochemical testing. Regarding statistical methodology, we were unable to perform multivariable regression or calculate effect sizes due to sample size constraints and data structure. In addition, there is potential for residual confounding from treatment factors (e.g. extent of resection, adjuvant radiation, and medication exposure), which are not accounted for by the baseline PANOMEN-3 grade. While we analyzed residual tumor, we could not account for specific intraoperative nuances, such as pituitary stalk manipulation or tumor consistency, which might have influenced immediate endocrine outcomes. Furthermore, regarding the composition of the nonoperative cohort, it is important to note that while the majority (60%) were non-functioning adenomas managed by surveillance, the functioning subset consisted almost exclusively of prolactinomas (97.2%). Therefore, while our results are applicable to conservatively managed non-functioning adenomas, findings regarding nonoperatively managed functioning tumors should be interpreted as specific to prolactinomas.

We propose incorporating PANOMEN-3 at the initial assessment to tier endocrine surveillance. Patients with grades 2–3, especially those with baseline hypopituitarism or large adenomas, warrant structured, interval screening for adrenal and thyroid deficiency, both postoperatively and during conservative care.

In conclusion, our findings extend the utility of PANOMEN-3 beyond predicting tumor behavior, establishing it as a valuable prognostic tool for secondary adrenal and thyroid dysfunction. With risk largely driven by baseline hypopituitarism and adenoma size, the score provides a simple clinical framework to guide the intensity of endocrine follow-up, identifying high-risk patients who require close monitoring while supporting de-escalation for those with low-grade scores.

## Declaration of interest

The authors declare no potential conflicts of interest with respect to the research, authorship, or publication of this article.

## Funding

This work did not receive any specific grant from any funding agency in the public, commercial, or not-for-profit sector.

## Author contribution statement

AN, GA, MAN, and SZ were responsible for the design of the study and data acquisition. AN, GA, and SZ contributed to the analysis and the interpretation of data, drafted the manuscript, and revised it critically for important intellectual content. MAN and GS contributed to the analysis and the interpretation of data and revised the manuscript critically for important intellectual content. All authors read and approved the final manuscript.

## Ethical statement

The study received ethical approval from Rambam Health Care Campus Institutional Review Board (protocol RMB-D-0322-24; September 23, 2024).

## Data availability

The raw data supporting the conclusions of this article will be made available by the authors, without undue reservation.
